# Effect of antibiotic use for acute bronchiolitis on new-onset asthma in children

**DOI:** 10.1038/s41598-018-24348-5

**Published:** 2018-04-17

**Authors:** I.-Lun Chen, Hsin-Chun Huang, Yu-Han Chang, Hsin-Yi Huang, Wei-Ju Yeh, Ting-Yi Wu, Jau-Ling Suen, San-Nan Yang, Chih-Hsing Hung

**Affiliations:** 1 Department of Pediatrics, Kaohsiung Chang Gung Memorial Hospital, Chang Gung University, College of Medicine, Kaohsiung City, Taiwan; 2 Graduate Institute of Medicine, College of Medicine, Kaohsiung Medical University, Kaohsiung City, Taiwan; 3 Teaching and Research Center of Kaohsiung Municipal Hsiao-Kang Hospital, Kaohsiung City, Taiwan; 4 Department of Pediatrics, E-DA Hospital and School of Medicine, College of Medicine, I-Shou University, Kaohsiung, Taiwan; 5 Department of Pediatrics, Kaohsiung Medical University Hospital, Kaohsiung, Taiwan; 6 Department of Pediatrics, Faculty of Pediatrics, College of Medicine, Kaohsiung Medical University, Kaohsiung City, Taiwan; 7 Department of Pediatrics, Kaohsiung Municipal Hsiao-Kang Hospital, Kaohsiung City, Taiwan; 8 Research Center for Environmental Medicine, Kaohsiung Medical University, Kaohsiung, Taiwan

## Abstract

Early-life use of antibiotics is associated with asthma. We examined the effect of antibiotic use for early-life bronchiolitis on the development of new-onset asthma in children from Taiwan between 2005 and 2010. Data were from the National Health Insurance Research Database 2010, and diseases were coded using the International Classification of Disease (ICD). We classified the patients, all of whom had bronchiolitis, as having asthma or not having asthma. Asthma was diagnosed using ICD criteria and by use of an inhaled bronchodilator and/or corticosteroid twice in one year. We identified age at asthma onset, sex, residential area, history of atopy and NSAID use, age at first use of antibiotics, and the specific antibiotic, and adjusted for these factors using conditional logistic regression analysis. Among all individuals, there was a relationship between risk of new-onset asthma with use of a high dose of an antibiotic (adjusted odds ratio [aOR] = 3.33, 95% confidence interval [CI] = 2.67–4.15). Among the different antibiotics, macrolides (aOR = 2.87, 95% CI = 1.99–4.16), and azithromycin specifically (aOR = 3.45, 95% CI = 1.62–7.36), had the greatest effect of development of asthma.

## Introduction

Environmental and genetic factors play important roles in the development of asthma. In particular, the “hygiene hypothesis” proposes that growing up in a hygienic environment, with reduced exposure to microbes, promotes T-helper type 2 (Th2) immunity^1^. The association of early-life use of antibiotics with wheezing symptoms and allergic rhinoconjunctivitis in childhood, and the lack of adverse effects of antibiotics on atopic diseases in childhood among infants who live in rural environments, are consistent with this hypothesis^2,3^.

Among babies younger than 1 year-old, acute bronchiolitis (a lower respiratory tract infection) is the leading cause of hospitalization due to recurrent wheezing episodes, and a history of bronchiolitis during infancy is associated with an increased risk for development of asthma^4^. Infants hospitalized with bronchiolitis due to respiratory syncytial virus (RSV) infection have a 2 to 3-fold increased risk of developing asthma later in childhood^5^. Another study also demonstrated that rhinovirus-induced wheezing within the first 3 years of life was associated with a nearly 10-fold increased risk for development of asthma by age six^6^.

Although most cases of acute bronchiolitis are due to viral infections, antimicrobial drugs may be used if there is suspected co-infection by bacteria or for their anti-inflammatory effects. In fact, a high incidence of bacterial co-infection was reported in children with severe bronchiolitis who required mechanical ventilation^7^. Bacterial PCR of blood samples showed that 10% of patients with acute bronchiolitis may have bacteremia, and the major species are *Haemophilus influenza* and *Streptococcus pneumonia*^8^. However, use of antibiotics early in life and a history of bronchiolitis increase the risk for asthma in young adolescents^9^. Other studies reported that polymorphisms of Toll-like receptor 4 might modify the effect of environmental factors, such as use of antibiotics, on the development of asthma^10,11^. Thus, reducing the use of antibiotics and prevention of bronchiolitis during infancy may prevent the development of asthma, especially in genetically susceptible subjects.

There is strong evidence of an association between acute bronchiolitis and asthma, but it is unclear whether this is a causal relationship. Although antibiotic use in early life increases the risk of asthma, the effect of antibiotic use by children for treatment of early-life bronchiolitis on the development of new-onset asthma is unknown. The aim of this study, which used the Taiwan National Health Insurance Research Database (NHIRD) 2010 database, was to assess the relationship of early-life antibiotic use for bronchiolitis with new-onset asthma in children.

## Methods

### Data source

This study examined claims data from the NHIRD, which includes the medical records of 99% of the 23.74 million residents of Taiwan, and 97% of Taiwanese hospitals and clinics who have contracts with the National Health Research Institute in Taiwan. The NHIRD contains comprehensive healthcare information, including demographic data of insured individuals, dates of clinical visits, diagnostic codes, and prescription details. All data in this study were from the NHIRD 2010, and all diseases were classified using the International Classification of Disease, Ninth Revision, Clinical Modification (ICD-9-CM). Patients with records indicating a diagnosis of bronchiolitis (ICD-9:466.19) during the first 2 years of life from 2000 to 2010 were included. New-onset asthma was diagnosed using the criteria of ICD-9:493 and receipt of selective beta-2 agonists and/or inhaled corticosteroid treatments twice within one year from age 2 to 18 years-old. This study was approved by the Institutional Review Board of Kaohsiung Medical University (KMUHIRB-E (I)-20150168). All methods were performed in accordance with the relevant guidelines and regulations.

### Use of antibiotics

The use of antibiotics was obtained from the outpatient prescription database, which listed the name of the drug and had data on dosage, date, and duration of prescription. To investigate the effect of antibiotic dosage, the cumulative defined daily dose (DDD) was calculated. Antibiotic use was defined as the prescription of at least one antibiotic (anatomical therapeutic chemical [ATC] code J01*) in the 5 years before onset of asthma. We analyzed data on 3 individual common classes of antibiotics—penicillins (ATC code J01C*), cephalosporins (ATC code J01D*), and macrolides (ATC code J01F*). Other types of antibiotics (quinolones, sulphonamides, glycopeptides, lincosamides) were analyzed separately.

### Potential confounders

The potential confounders of asthma included urban residence, allergic rhinitis (ICD9-CM: 477.8, 477.9), atopic dermatitis (ICD9-CM: 691.8), chronic rhinitis (ICD9-CM:472.0), acute sinusitis (ICD9-CM:461), gastroesophageal reflux disease (GERD) (ICD9-CM:530.81), and use of a nonsteroidal anti-inflammatory drug (NSAID) in the 120 days before the onset of asthma.

### Statistical analysis

Pearson’s chi-square test or Fisher’s exact test and the independent *t* test were used to evaluate the significance of differences of categorical and continuous variables in patients with and without asthma. Conditional logistic regression analysis was used to calculate the association between asthma and antibiotic use. As recommended by the World Health Organization (WHO), cumulative DDD was used to quantify the cumulative dose of antibiotics, and the categories were “low dose”, “moderate dose”, and “high dose”. Several co-variables, including age, sex, residential area, history of atopy, and use of NSAIDs, were included in the statistical model. The effect of antibiotic class was also analyzed by adjusting for co-variables and type of antibiotic, except for penicillins, cephalosporins, and macrolides. Odds ratios (ORs), adjusted ORs (aORs) and 95% confidence intervals (CIs) were calculated to show the risk for development of asthma. We further used false discovery correction to evaluate each variables and Benjamini-Hochberg P-values were showed in tables. All statistical analyses were performed using SAS 9.3, and *p* value below 0.05 was considered significant.

## Results

After propensity score matching, the non-asthma and asthma groups each had 2082 children (Table 1). Due to matching, these 2 groups had similar distributions of age, sex, urbanization of residence, comorbidities, and use of NSAIDs.

**Table 1:** The characteristics of non-asthma and asthma group (N = 4,164).

	Non-asthma group (n = 2082)		Asthma group (n = 2082)		p value
	N	(%)	N	(%)	
Age, mean ± SD	3.32 ± 1.25		3.34 ± 1.26		0.497
Gender					
Female	921	(44.2)	907	(43.6)	0.662
Male	1161	(55.8)	1175	(56.4)	
Resident urbanization					
Urban	463	(22.2)	451	(21.7)	0.807
Suburban	806	(38.7)	818	(39.3)	
Rural	813	(39.0)	813	(39.0)	
Comorbidities					
Allergic rhinitis	644	(30.9)	668	(32.1)	0.423
Atopic dermatitis	466	(22.4)	456	(21.9)	0.709
Chronic rhinitis	309	(14.8)	322	(15.5)	0.574
Acute sinusitis	1615	(77.6)	1628	(78.2)	0.627
GERD	31	(1.5)	23	(1.1)	0.273
Medication					
NSAIDs	482	(23.2)	471	(22.6)	0.685

Univariable and multivariable analysis indicated significant associations between use of any antibiotic and cumulative DDDs of any antibiotic with development of new-onset asthma (aOR = 2.10, 95% CI = 1.75–2.20) (Table 2). Analysis of the different classes of antibiotics also indicated significant associations between the cumulative DDDs for penicillins, cephalosporins, and macrolides with the development of new onset asthma. Use of other antibiotics (quinolones, sulphonamides, glycopeptides, lincosamides) had no effect on the development of asthma.

**Table 2:** The association between antibiotics cumulative DDDs exposure categories and the risk of new-onset asthma in ever-diagnosed bronchiolitis population (n = 4,164).

Antibiotic use	Non-asthma group (n = 2082)		Asthma group (n = 2082)		Unadjusted Model		Adjusted Model	
	N	(%)	N	(%)	OR (95% CI)	p value	OR (95% CI)	p value
J01 antibiotic*								
No	419	(20.1)	233	(11.2)	1.00		1.00	
Yes	1663	(79.9)	1849	(88.8)	2.00(1.68–2.38)	<0.001	2.10(1.75–2.52)	<0.001
Cumulative DDDs								
<3.50	648	(31.1)	557	(11.2)	1.55(1.27–1.88)	<0.001	1.64(1.35–2.01)	<0.001
3.50–10.00	544	(26.1)	589	(26.8)	1.95(1.60–2.37)	<0.001	2.23(1.81–2.74)	<0.001
>10.00	471	(22.6)	703	(33.8)	2.68(2.20–3.27)	<0.001	3.33(2.67–4.15)	<0.001
p for trend						<0.001		<0.001
J01C Penicillin**	1712		1701					
No	419	(24.5)	233	(13.7)	1.00		1.00	
Yes	1293	(75.5)	1468	(86.3)	2.04(1.71–2.44)	<0.001	1.77(1.43–2.20)	<0.001
Cumulative DDDs								
<3.00	572	(33.4)	505	(29.7)	1.59(1.30–1.94)	<0.001	1.48(1.18–1.87)	0.001
3.00–7.55	351	(20.5)	412	(24.2)	2.11(1.70–2.62)	<0.001	2.00(1.55–2.57)	<0.001
>7.55	370	(21.6)	551	(32.4)	2.68(2.18–3.30)	<0.001	2.62(2.02–3.39)	<0.001
p for trend						<0.001		<0.001
J01D Cephaloporins**	1549		1530					
No	419	(27.0)	233	(15.2)	1.00		1.00	
Yes	113	(73.0)	1297	(84.8)	2.06(1.73–2.47)	<0.001	1.67(1.32–2.11)	<0.001
Cumulative DDDs								
<1.25	420	(27.1)	446	(29.2)	1.91(1.55–2.35)	<0.001	1.61(1.26–2.06)	<0.001
1.25–3.31	351	(22.7)	401	(26.2)	2.05(1.66–2.55)	<0.001	1.71(1.31–2.23)	<0.001
>3.31	359	(23.2)	450	(29.4)	2.25(1.82–2.77)	<0.001	1.85(1.40–2.45)	<0.001
p for trend						<0.001		<0.001
J01F Macrolides**	970		978					
No	419	(43.2)	233	(23.8)	1.00		1.00	
Yes	551	(56.8)	745	(76.2)	2.43(2.00–2.95)	<0.001	2.02(1.48–2.76)	<0.001
Cumulative DDDs								
<1.50	240	(24.7)	234	(23.9)	1.75(1.38–2.23)	<0.001	1.57(1.12–2.20)	0.009
1.50–3.00	140	(14.4)	205	(21.0)	2.63(2.01–2.44)	<0.001	2.38(1.66–3.41)	<0.001
>3.00	171	(17.6)	306	(31.3)	3.22(2.56–4.12)	<0.001	2.87(1.99–4.16)	<0.001
p for trend						<0.001		<0.001
J01 Others**	538		378					
No	419	(77.9)	233	(61.6)	1.00		1.00	
Yes	119	(22.1)	145	(38.4)	2.19(1.64–2.93)	<0.001	1.40(0.71–2.75)	0.466
Cumulative DDDs								
<1.00	45	(8.4)	54	(14.3)	2.16(1.41–3.31)	<0.001	1.40(0.67–2.97)	0.466
1.00–2.00	30	(5.6)	35	(9.3)	2.10(1.26–3.51)	<0.001	1.34(0.61–2.96)	0.466
>2.00	44	(8.2)	56	(14.8)	2.29(1.50–3.51)	<0.001	1.45(0.67–3.12)	0.466
p for trend						<0.001		0.485

*Adjusted age, gender, resident urbanization, other comorbidities and medication.

**Adjusted age, gender, resident urbanization, other comorbidities, medication and other subtype antibiotics.

***p value was performed by Benjamini-Hochberg P-values.

Overall, individuals who first received an antibiotic by age 2 (aOR = 1.67, 95% CI = 1.27–2.19) or by age 3 (aOR = 1.99, 95% CI = 1.29–3.09) were more significantly more likely to develop new-onset asthma (Table 3). Age-stratified analysis indicated that children who were diagnosed with new-onset asthma at age 3–4 were significantly more likely to have used antibiotics by age 2 (aOR = 1.78, 95% CI = 1.32–2.39) and by age 3 (aOR = 2.95, 95% CI = 1.72–5.07). This relationship was not significant for children who had new-onset asthma when older than age 4.

**Table 3:** The association between childhood antibiotics exposure and the risk of new-onset asthma in ever-diagnosed bronchiolitis population (n = 4164).

Exposure age	Non-asthma group		asthma group		Model I		
	N	(%)	N	(%)	Adjusted OR (95% CI)	p value	p value*
Overall	2082		2082				
No exposure	426	(20.5)	256	(12.3)	1.00		
<=1 years old	1371	(65.9)	1396	(67.1)	1.01(0.78–1.30)	0.955	0.955
2 years old	242	(11.6)	361	(17.3)	1.67(1.27–2.19)	<0.001	<0.001
3 years old	43	(2.1)	69	(3.3)	1.99(1.29–3.09)	0.002	0.003
3–4 y/o	1735		1721				
No exposure	400	(23.1)	239	(13.9)	1.00		
<=1 years old	1130	(65.1)	1128	(65.5)	0.90(0.68–1.19)	0.463	0.463
2 years old	182	(10.5)	300	(17.4)	1.78(1.32–2.39)	<0.001	<0.001
3 years old	23	(1.3)	54	(3.1)	2.95(1.72–5.07)	<0.001	<0.001
>4 y/o	347		361				
No exposure	26	(7.5)	17	(4.7)	1.00		
<=1 years old	241	(69.5)	268	(74.2)	1.26(0.59–2.71)	0.552	0.871
2 years old	60	(17.3)	61	(16.9)	1.18(0.52–2.64)	0.696	0.871
3 years old	20	(5.8)	15	(4.2)	0.92(0.35–2.43)	0.871	0.871

Adjusted age, gender, resident urbanization, other comorbidities and medication.

*Benjamini-Hochberg P-values.

**Figure 1:**
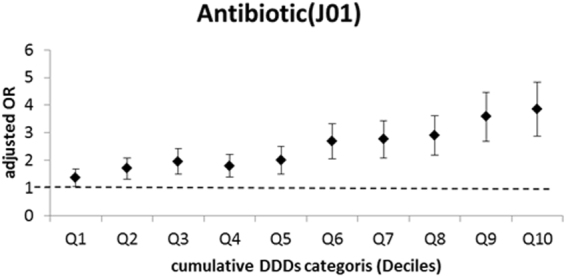
The relationship of cumulative DDDs of antibiotics and new-onset asthma.

Separation of patients into 3 groups based on cumulative DDDs of antibiotic used indicated the frequency of overall antibiotic use in the asthma group was 11.2% (low dose), 26.8% (moderate dose), and 33.8% (high dose) (Table 2). There was also dose-dependent relationship of cumulative DDDs of antibiotic use with the aOR for new-onset asthma (Fig. 1).

Age-stratified analysis indicated that children younger than 5 who used any antibiotic (aOR = 2.07, 95% CI = 1.82–2.50), a penicillin (adjusted OR 1.73, 95% CI 1.73–2.17), a cephalosporin (adjusted OR 1.68, 95% CI 1.31–2.14), or a macrolide (adjusted OR 1.97, 95% CI 1.41–2.75) had an increased risk for development of new-onset asthma (Table 4). There was no such effect for children older than 5.

**Table 4:** The association between the species of antibiotics and the risk of new-onset asthma in ever-diagnosed bronchiolitis population by age-stratified analysis (n = 4,164).

Antibiotic use	Non-asthma group		Asthma group		Unadjusted Model			Adjusted Model		
	N	(%)	No	(%)	OR (95% CI)	p value	p value***	OR (95% CI)	p value	p value***
Less than 5 years old										
J01 Antibiotics*	1335	(76.9)	1496	(86.9)	1.99(1.67–2.38)	<0.001	<0.001	2.07(1.82–2.50)	<0.001	<0.001
J01C Penicillin**	1007	(71.6)	1155	(83.7)	2.04(1.70–2.45)	<0.001	<0.001	1.73(1.38–2.17)	<0.001	<0.001
J01D Cephaloporins**	855	(68.1)	1001	(81.6)	2.08(1.73–2.51)	<0.001	<0.001	1.68(1.31–2.14)	<0.001	<0.001
J01F Macrolides**	408	(50.5)	558	(71.3)	2.43(1.98–2.99)	<0.001	<0.001	1.97(1.41–2.75)	<0.001	<0.001
J01 Others**	83	(172)	94	(29.5)	2.01(1.44–2.82)	<0.001	<0.001	1.30(0.64–2.65)	0.474	0.474
More than 5 years old										
J01 Antibiotics*	328	(94.5)	353	(97.8)	2.56(1.10–5.92)	0.028	0.029	2.85(1.21–6.69)	0.016	0.032
J01C Penicillin**	286	(93.8)	313	(97.5)	2.60(1.12–6.03)	0.026	0.029	2.53(0.98–6.53)	0.055	0.0.92
J01D Cephaloporins**	275	(93.5)	296	(97.4)	2.56(1.10–5.94)	0.029	0.029	1.85(0.70–4.91)	0.218	0.267
J01F Macrolides**	143	(88.3)	187	(95.9)	3.11(1.32–7.30)	0.009	0.015	2.72(0.87–8.53)	0.085	0.121
J01 Others**	36	(65.5)	51	(86.4)	3.37(1.33–8.53)	0.011	0.016	5.17(0.33–80.22)	0.240	0.267

*Adjusted age, gender, resident urbanization, other comorbidities and medication.

**Adjusted age, gender, resident urbanization, other comorbidities, medication and other subtype antibiotics.

***Benjamini-Hochberg P-values.

Further analysis of the cumulative DDDs of specific penicillins and macrolides indicated that a high doses of amoxicillin (aOR = 2.61 95% CI = 2.02–3.38), erythromycin (aOR = 2.44 95% CI = 1.61–3.37), and azithromycin (aOR = 3.45 95% CI = 1.62–7.36) were associated with increased risk for development of new-onset asthma (Table 5).

**Table 5:** The association between the subtype of antibiotics and the risk of new-onset asthma in ever-diagnosed bronchiolitis population (n = 4164).

Antibiotic use	Non-asthma group		Asthma group		Unadjusted Model			Adjusted Model		
	N	(%)	N	(%)	OR (95% CI)	p value	p value*	OR (95% CI)	p value	p value*
J01C Penicillin										
J01CA Amoxicillin	1712		1701							
No	419	(24.5)	233	(13.7)	1.00			1.00		
Yes	1293	(75.5)	1468	(86.3)	2.04(1.71–2.44)	<0.001	<0.001	1.77(1.43–2.20)	<0.001	<0.001
Cumulative DDDs										
<3.00	573	(33.5)	506	(29.7)	1.59(1.30–1.94)	<0.001	<0.001	1.49(1.18–1.87)	0.001	0.002
3.00–7.50	338	(19.7)	394	(23.2)	2.10(1.69–2.60)	<0.001	<0.001	1.99(1.54–2.56)	<0.001	<0.001
>7.50	382	(22.3)	568	(33.4)	2.67(2.18–3.29)	<0.001	<0.001	2.61(2.02–3.38)	<0.001	<0.001
J01CA01 Ampicillin	467		293							
No	419	(89.7)	233	(79.5)	1.00			1.00		
Yes	48	(10.3)	60	(20.5)	2.25(1.49–3.39)	<0.001	<0.001	0.97(0.41–2.31)	0.948	0.948
Cumulative DDDs										
<0.50	24	(5.1)	28	(9.6)	2.10(1.19–3.70)	0.011	0.013	1.04(0.41–2.62)	0.943	0.948
0.50–1.00	5	(1.1)	10	(3.4)	3.60(1.22–10.65)	0.021	0.022	1.82(0.43–7.69)	0.413	0.508
>1.00	19	(4.1)	22	(7.5)	2.08(1.10–3.93)	0.023	0.023	0.68(0.22–2.09)	0.506	0.578
J01F Macrolides										
J01FA10 Azithromycin	544		462							
No	419	(77.0)	233	(50.4)	1.00			1.00		
Yes	125	(23.0)	229	(49.6)	3.29(2.51–4.32)	<0.001	<0.001	2.53(1.41–4.53)	0.002	0.003
Cumulative DDDs										
<1.67	49	(9.0)	48	(10.4)	1.76(1.15–2.71)	0.010	0.012	1.43(0.72–2.82)	0.306	0.408
1.67–2.50	48	(8.8)	109	(23.6)	4.08(2.81–5.94)	<0.001	<0.001	3.39(1.80–6.38)	<0.001	<0.001
>2.50	28	(5.1)	72	(15.6)	4.62(2.91–7.36)	<0.001	<0.001	3.45(1.62–7.36)	0.001	0.002
J01FA01 Erythromycin	840		767							
No	419	(49.9)	233	(30.4)	1.00			1.00		
Yes	421	(50.1)	534	(69.6)	2.28(1.86–2.80)	<0.001	<0.001	2.00(1.40–2.85)	<0.001	<0.001
Cumulative DDDs										
<1.50	202	(24.0)	218	(28.4)	1.94(1.51–2.49)	<0.001	<0.001	1.77(1.21–2.59)	0.003	0.004
1.50–3.00	80	(9.5)	107	(14.0)	2.41(1.73–3.35)	<0.001	<0.001	2.18(1.41–3.38)	<0.001	<0.001
>3.00	139	(16.5)	209	(27.2)	2.70(2.07–3.53)	<0.001	<0.001	2.44(1.61–3.37)	<0.001	<0.001

Adjusted age, gender, resident urbanization, other comorbidities, medication and other subtype antibiotics.

*Benjamini-Hochberg P-values.

## Discussion

Our results indicate that early use of antibiotics in children with acute bronchiolitis is significantly associated with the development of new-onset asthma. In addition, our comparison of the effects of individual antibiotics indicated that use of high-dose azithromycin had the greatest impact on the development of new-onset asthma.

Bronchiolitis is the most common reason for hospitalization of infants and young children. The respiratory syncytial virus (RSV) and rhinovirus are the two most common pathogens in acute bronchiolitis^12^, and infection with either virus increases the risk for allergic diseases. Previous research reported that azithromycin can reduce wheezing episodes during RSV-mediated bronchiolitis due to its anti-inflammatory effect^13^. In Taiwan, antibiotics may be prescribed to children younger than age 2 for treatment of acute bronchiolitis, in an effort to prevent bacterial supra-infection or to reduce wheezing. In recent decades, there has been a high rate of antibiotic use by children with lower respiratory tract infections, especially during infancy^14^. Use of antibiotics during infancy increases the risk of childhood asthma^15,16^. Moreover, when pregnant women use antibiotics in the third trimester of pregnancy, their children have a slightly increased risk of asthma when in preschool^17^.

Asthma is a multifactorial disease, and genetic and environmental factors can affect the risk for developing asthma. The diagnosis of asthma early in a child’s life is particularly difficult, because cooperation during functional testing is difficult and frequent wheezing episodes may occur due to respiratory infections. Thus, clinicians diagnose asthma in young children based on symptoms using a differential diagnosis, and response to anti-asthmatic medications. Over-diagnosed asthma is sometimes found in Taiwan due to the policy of national health insurance and the diagnosis of real asthma is difficult in young age. In our study, we examined children with diagnoses of asthma who received selective beta-2 agonists and/or inhaled corticosteroids twice within 1 year to discriminate asthma from transient wheezing.

During gestation, the immune phenotype is skewed toward Th2 cells, which protect the developing embryo from the maternal immune response and thereby prevent miscarriage and premature labor^18^. Thus, Th2-associated chemokines—CCL17 and CCL22—are highest in neonates, and decline gradually during the following 2 years^19^. The presence of environmental stimuli during pregnancy and early life may potentially affect Th1/Th2 immunity and programming by affecting chromatin remodeling processes^20^. In particular, Th2 dominance can shift to Th1 dominance following exposure to infectious microorganisms. However, disruption of the normal Th1/Th2 realignment during infancy may lead to the development of atopic disease and allergy^20^.

Most interactions between the host immune system and the microbiome occur in the gut. Symbiotic or pathogenic microorganisms in the human gut can affect the immune response through gut-associated lymphoid tissues and Toll-like receptors, ultimately leading to various inflammatory disorders^21^. Antibiotic use during early life can alter the gut microbiota, and lead to inflammatory and allergic diseases. These adverse effects of antibiotic use diminish with age, based on the results of the present study (Table 4) and a previous cohort study, which reported that the risk of developing asthma in children was greater when they used antibiotics during the first year of life^15^.

No previous studies have examined the simultaneous presence of acute bronchiolitis and antibiotic use on the development of asthma in children. However, previous cohort studies in Europe and Canada reported associations in the use of ampicillin, cephalosporin, and macrolides with the development of asthma, and positive correlations of the accumulative antibiotic dose with asthma^9,16^. We found that use of azithromycin, relative to other antibiotics, led to a greater risk of new-onset asthma among children younger than age 5 who ever had acute bronchiolitis. A previous multi-center cohort study reported that macrolide use during the first year of life was associated with an increased risk for wheezing by 36 months^9^. Macrolides have anti-bacterial effects and immunomodulatory properties, in that they inhibit T-cell activation, down-regulate pro-inflammatory cytokines, decrease mucus synthesis, and promote apoptosis of inflammatory cells in the bronchial epithelium^22,23^. In addition, macrolides can reduce the frequency of exacerbations in eosinophilic asthma^24^, and is given to infants who have wheezing symptoms resulting from acute bronchiolitis. However, the mechanism by which macrolide administration during infancy induces new-onset asthma during childhood is unknown. Further studies of this important topic are necessary.

Our study had several limitations in that we did not consider confounding by socioeconomic status, exposure to second-hand smoke, and the presence of atopic diseases in parents and siblings. Moreover, definitive diagnosis of asthma in preschool children is difficult, and under-diagnosis may have led to the inappropriate exclusion of certain children.

In conclusion, our results provide evidence that infants with acute bronchiolitis who use a penicillin, a cephalosporin, or a macrolide—especially azithromycin—have an increased risk for developing new-onset asthma during childhood. These results are consistent with the predictions of the hygiene hypothesis.
